# Reporting of thermography parameters in biology: a systematic review of thermal imaging literature

**DOI:** 10.1098/rsos.181281

**Published:** 2018-12-05

**Authors:** Michael J. M. Harrap, Natalie Hempel de Ibarra, Heather M. Whitney, Sean A. Rands

**Affiliations:** 1 School of Biological Sciences, University of Bristol, Bristol BS8 1TQ, UK; 2 Centre for Research in Animal Behaviour, School of Psychology, University of Exeter, Exeter EX4 4QG, UK

**Keywords:** thermography, thermal camera, infrared, emissivity, temperature measurement

## Abstract

Infrared (IR) thermography, where temperature measurements are made with IR cameras, has proven to be a very useful and widely used tool in biological science. Several thermography parameters are critical to the proper operation of thermal cameras and the accuracy of measurements, and these must usually be provided to the camera. Failure to account for these parameters may lead to less accurate measurements. Furthermore, the failure to provide information of parameter choices in reports may compromise appraisal of accuracy and replicate studies. In this review, we investigate how well biologists report thermography parameters. This is done through a systematic review of biological thermography literature that included articles published between years 2007 and 2017. We found that in primary biological thermography papers, which make some kind of quantitative temperature measurement, 48% fail to report values used for emissivity (an object's capacity to emit thermal radiation relative to a black body radiator), which is the minimum level of reporting that should take place. This finding highlights the need for life scientists to take into account and report key parameter information when carrying out thermography, in the future.

## 1. Introduction

Temperature is an important biological variable. It is a key influence on living organisms [1–8], and temperature can also be used as an indicator for metabolic activity [7,9–11], disease, injury and stress [12–16]. Temperature of organisms has been measured using thermocouples [17–19] or thermistors [20,21], though use of thermographic cameras has increased dramatically in recent years with improvement of the technology [14,22]. Thermographic cameras detect the radiation from all objects hotter the absolute zero, usually in the human invisible ‘thermal infrared band', wavelength range of 2–14 µm. These radiation measurements, along with thermography parameters that are input into the camera, can be used to estimate the temperature of an object. The main thermography parameter is the target object's emissivity, which is its capacity to radiate infrared (IR) radiation relative to a black body radiator at the same temperature. Other parameters used are information about the environment in which measurements are taking place: IR reflections, distance between camera and target, environmental temperature and environmental humidity [22–24]. Thermography has a number of benefits when compared with other temperature measurement methods such as thermocouples [25,26]. Firstly, in contrast to thermocouples and thermistors with individual contact points, it is easier with thermal cameras to measure the changes of temperature with high spatial resolution, across a target or simultaneously in several targets [14,27–29]. Secondly, it responds quickly to changes allowing monitoring of subjects that are moving or might change temperature quickly [27,30]. Lastly, and possibly most importantly to biologists, it is non-contact [22,23,25]; this is important because attempting contact measurements with biological subjects may disturb or damage the subject, or in more delicate applications disrupt temperature distributions. Using a non-contact technique also means temperature measurements can be made on more distant targets [31–33].

Infrared thermography is a valuable tool for biologists and has been widely applied for temperature measurements [14,22,25,26,29,34]. However, doubt has been expressed over how well biologists understand and use these tools [22]. Understanding of how thermal cameras estimate the temperature of objects requires an understanding of the thermography parameters that must be entered into the camera. Here, we will discuss these parameters and assess how they are reported in the biological literature using a systematic literature review. Correct reporting is important, as it is both vital for ensuring repeatability of a thermographic study, and allows a reader to evaluate the correctness of a reported result. By reviewing how often thermographic parameters are reported, we can evaluate how well life scientists appear to understand thermography. Based on our findings, we will provide advice for biological thermographers and highlight common mistakes that can be easily avoided in future work.

## 2. Background information

### 2.1. Principles of thermography

All objects of a temperature above absolute zero emit electromagnetic radiation. Increased temperature leads to increased levels of radiation [35,36]. This radiation is usually within the thermal IR band, which is invisible to humans and has wavelength ranges between 0.8 and 14 µm [22–24]. However, once heated to a certain point, objects will begin to radiate more in the shorter wavelengths, including in the light spectrum visible to humans. Thermal cameras are equipped with IR-transmitting optics and arrays of sensors that are sensitive to portions of the thermal IR band [22–24]. The sensor readings are converted to radiometric units and colour-coded to generate false colour images that allow us to visualize thermal IR radiation that cannot be seen by the human eye. Most commercially available thermal cameras are sensitive to either mid-wave IR (2–5 µm) or long-wave IR (8–14 µm) [22–24]. These restrictions of wavelengths cameras are sensitive to are of the wavelengths of expected thermal radiation and those that provide high transmission (see below) through the atmosphere and camera optics [22–24].

The thermal radiation emitted by an object (*W*_obj_) is dependent on the object's temperature (*T*_obj_, measured in *K*) in accordance with the Stefan–Boltzmann formula [35,36]:

2.1 $$W_{\mathrm{obj}}=\varepsilon \cdot \sigma \cdot T_{\mathrm{obj}}^{4},$$

where *σ* is the Stefan–Boltzmann constant (*ca* 5.67 × 10^−8^ W m^−2^ K^−4^) and *ɛ* is the emissivity of the object. Emissivity is the capacity of an object to emit thermal radiation relative to a black body at the same temperature. A black body is a theoretical body which is non-transmissive and non-reflective, in other words completely absorbs any kind of incident electromagnetic radiation. Emissivity is represented as a fraction between 0 and 1, and black bodies have an *ɛ* of 1.

A thermal camera detects electromagnetic waves in the thermal IR band, and just like a regular human-visible light camera does not distinguish between emitted and reflected radiation. Like human-visible light, thermal radiation has to be transmitted through the atmosphere. Furthermore, the atmosphere itself emits thermal IR radiation [22–24]. Thus, when imaging a non-transmissive object through the air, the total radiation *W*_tot_ entering a thermal camera will be the sum of the emitted radiation of the object (*W*_obj_), the amount of radiation reflected off the object (*W*_ref_) and the amount of radiation emitted by the atmosphere (*W*_atm_):

2.2 $$W_{\mathrm{tot}}=W_{\mathrm{obj}}+W_{\mathrm{ref}}+W_{\mathrm{atm}}.$$

This means that the radiation-based image viewed through the camera does not necessarily indicate the focal object's temperature, and that some level of calibration of the raw radiation image is needed to account for these additional sources of radiation [24]. This uncalibrated thermal image is known as ‘apparent temperature'. *W*_obj_, *W*_ref_ and *W*_atm_ are each influenced by the transmissivity of the atmosphere between the object and camera, *τ*_atm_, and can be calculated by:

2.3 $$W_{\mathrm{obj}}=\varepsilon \cdot \sigma \cdot \tau_{\mathrm{atm}}\cdot {\left(T_{\mathrm{obj}}\right)}^{4},$$

2.4 $$W_{\mathrm{ref}}=(1-\varepsilon )\cdot \sigma \cdot \tau_{\mathrm{atm}}\cdot {\left(T_{\mathrm{ref}}\right)}^{4}$$

2.5 $$\;\mathrm{and}\;W_{\mathrm{atm}}=\sigma \cdot (1-\tau_{\mathrm{atm}})\cdot {\left(T_{\mathrm{env}}\right)}^{4},$$

where *T_x_* refers to the temperature of *x* (*x* being the object, the environment or reflections). Note that the emissivity of the atmosphere equals (1 − *τ*_atm_), as objects can either emit, transmit or reflect radiation [23] and the atmosphere is non-reflective within the thermal IR band. Equations (2.3)–(2.5) can be substituted into equation (2.2) to give,

2.6 $$W_{\mathrm{tot}}=\varepsilon \cdot \sigma \cdot \tau_{\mathrm{atm}}\cdot {\left(T_{\mathrm{obj}}\right)}^{4}+(1-\varepsilon )\cdot \sigma \cdot \tau_{\mathrm{atm}}\cdot {\left(T_{\mathrm{ref}}\right)}^{4}+\sigma \cdot (1-\tau_{\mathrm{atm}})\cdot {\left(T_{\mathrm{env}}\right)}^{4},$$

which can be reorganized

2.7 $$T_{\mathrm{obj}}=4\sqrt{\frac{W_{\mathrm{tot}}-(1-\varepsilon )\cdot \;\tau_{\mathrm{atm}}\cdot \sigma \;\cdot {\left(T_{\mathrm{ref}}\right)}^{4}-(1-\tau_{\mathrm{atm}})\;\cdot \sigma \;\cdot \;{\left(T_{\mathrm{env}}\right)}^{4}}{\varepsilon \cdot \tau_{\mathrm{atm}}\cdot \sigma }},$$

to give temperature estimates of the object of interest.

The calculation in equation (2.7) is normally carried out by the camera itself, or related software (e.g. FLIR tools [37]) after the image has been captured [24]. Equation (2.7) identifies several parameter inputs required by the camera, or software, to accurately measure the temperature of the object. These must be applied to images before measurements of temperature are taken from them, using the camera or related software. However, several of these parameter inputs are dependent on the time of image capture. Thus, although they can be applied to images afterwards, they must be measured at the time of thermograph capture. A checklist summary of the requirements for obtaining the most accurate thermographic temperature measurements and how the required timings influence protocol, is provided in table 1. The best quality thermographic measurements require accurate estimates of these parameter inputs in addition to correct use of camera optics in terms of image focus [23,24].

**Table 1.** A checklist for accurate thermographic temperature measurements. The six aspects needed for accurate thermographic temperature measurements are listed, as well as where the timing of such aspects should be considered in experimental protocols. Note that the requirements, although all contributing to maximizing accuracy, do not influence accuracy equally. This checklist assumes thermography is not being carried out through a thermal IR transmissive window. It is very unlikely that researchers conducting biological thermography would need to use a transmissive window, but if this is the case further considerations must be made (see [24]).

aspect	ideal requirements	timing considerations
quality thermographic image capture	a well-focused, unobscured image of target organism or tissues	Thermograph image focus and content cannot be altered after capture. Image contrast and appearance in terms of temperature scales can be altered and are not important for temperate measurements, although they can aid with obtaining good image focus.
emissivity (*ɛ*) estimate	a measurement of emissivity from the same object being thermographed	Emissivity can normally be applied to images after capture. It does not necessarily need to be known at the time of image capture but needs to be obtained and applied to an image before measurements are taken from images.
reflected temperature (*T*_ref_) measurement	a measurement of reflected temperature off the thermography target	Measurement of reflected temperature should be made simultaneously with each thermographic image capture. More practically, mirrors require *T*_ref_ measurements to be made immediately after image capture. Reflected temperature can then normally be applied to the relevant thermograph images after capture.
environmental temperature (*T*_env_) measurements	a measurement of the temperature of the environment where the thermal image was captured	Should be made simultaneously with image capture. Environmental temperature can then normally be applied to the relevant thermograph images after capture.
environmental relative humidity (*rh*) measurements	a measurement of the relative humidity of the environment where the thermal image was captured	Measurements should be made simultaneously with image capture. Environmental relative humidity can then normally be applied to the relevant thermograph images after capture. This is used by the camera or software to calculate *τ*_atm_.
distance between the camera and thermography target (*d*)	a measure of distance between the camera and thermography target	This should be either controlled, and therefore known, or measured after image capture. As long as positions are noted, this measurement does not need to occur right away. This is used by the camera or software to calculate *τ*_atm_.

### 2.2. Emissivity

Object emissivity, *ɛ*, alternatively called ‘emittance’, ‘emission' or ‘emission coefficient', is a proportion (bound between 0 and 1) that represents the capacity of an object to radiate thermal IR radiation relative to a black body at the same temperature [22–24]. An emissivity of 1 treats the target object as a black body. Objects with high emissivity have temperatures that align closely with apparent temperature, while the total radiation entering a thermal camera (*W*_tot_) when observing a low emissivity object will be influenced more strongly by reflected IR radiation (equation (2.6)).

Emissivity can be measured using several methods, usually involving comparing the radiation from the object with that of a known emissivity of the same temperature [24]. This can be achieved by coating part of the object in something of known emissivity and heating the object evenly. Here, a true measurement of the object temperature can be made with the thermal camera, and the emissivity parameter can then be adjusted until matching estimates of temperature are achieved on the uncoated parts of the object [10,38,39]. Often such coating is difficult on biological subjects, and heating live subjects evenly can be difficult and unethical. Although estimates could be carried out using dead subjects, where suitable and ethically obtainable [22,40]. Alternatively, if the objects' temperature is known through another temperature measurement method, emissivity can be calculated by rearranging equation (2.7) [38,41–43].

Inaccurate estimates of emissivity have the largest influence on the accuracy of temperature measurements [22,23]. As seen in equation (2.7), changing emissivity changes the portion of *W*_tot_ taken to be from the object itself as opposed to from other sources and can lead to misjudgements in the contribution of reflections to *W*_tot_ relative to the object radiation. Emissivity has a direct effect on the temperature the object is estimated to have when emitting a given amount of radiation. Therefore, information on emissivity of the object is key for thermographic measurements.

Emissivity is normally high in biological tissues, approximately 0.9 or higher (e.g. [22,25]). This has the benefit that the impacts on inaccurate emissivity measurements are reduced when compared to low emissivity objects (see equation (2.7)). An inaccurate but still high emissivity value, assuming the target's true emissivity is, in fact, high, will cause smaller levels of inaccuracy then similar inaccuracy in low emissivity targets [22,23]. However, such impacts are not removed entirely. Emissivity is primarily influenced by the object's composition, and this can vary across different biological tissues. Emissivity can also be influenced by object properties such as geometry and surface structure [24]. As these can differ across and between different types of biological subjects [44,45], it is advised that when appropriate sources for emissivity values are not available, emissivity is measured on the tissues to be thermographed or estimated based on sources on a similar tissue.

### 2.3. Reflected temperature

Reflected temperature (*T*_ref_) is an estimate of the level of background radiation reflected off the thermography target object [22–24], and is frequently expressed as a temperature value. Reflected temperature can also be referred to as ‘reflected apparent temperature', ‘background radiation’, ‘reflected radiation from ambient sources'. Also, confusingly, simply ‘ambient' or ‘background temperature' can be used to describe reflected temperature [46–49]. Such terms for reflected temperature can be easily confused with environmental temperature (*T*_env_), and should be discouraged. It should be clearly stated what information is used to estimate reflected and environmental temperature in calculations. This is especially true as environmental temperature can be used as a reasonable estimate of reflected temperature [23].

There are several ways this value can be estimated alongside thermographic measurements. A mirrored surface [23,46], preferably a multidirectional mirror [38,43], placed on a plane with the thermography target can be used to measure *T*_ref_. Here *T*_ref_ is taken as the average apparent temperature of the mirror (achieved by setting the camera's emissivity to 1 and distance to 0). Practically speaking this normally involves taking a second thermograph of the target with the mirror placed in frame alongside it in the same plane immediately after measurements are taken. *T*_ref_ can then be calculated and applied to the initial image [38,46]. Alternatively, the environmental temperature is often a reasonable estimate of reflected temperature [23], as long as no sources of a large amount of light or heat are near the object. Such sources of heat and light may lead to reflected temperature differing from environmental temperature. Efforts can be taken to minimize sources of reflected temperature, such as shielding and repositioning the camera; however, an accurate measure of reflected temperature value still has to be entered into the camera, and how reflected temperature was estimated should still be reported. Reflected temperature should be measured simultaneously or immediately following thermographic measurements, as changes in conditions or positioning of objects can alter reflected temperature, as noted in table 1.

Inaccurate estimates of reflected temperature can lead to misjudgement of the amount of radiation coming from the target object and other sources. However, biological tissues have a high emissivity, so the contribution of reflected temperature to *W*_tot_ is usually small [22] within biological applications (see equation (2.7)). Usually, the best estimate of *T*_ref_ is achieved by measuring it along with each thermograph using a multidirectional mirror. This can be easier with stationary targets unlikely to move, such as plants. Similarly, in laboratory conditions multidirectional mirrors can be installed in such a way that *T*_ref_ measurements are taken simultaneously with target measurements (as in [43]). The use of mirrors and constant measurement of reflected temperature can be impractical in some experiments. Biological targets, particularly wild animals can be disturbed by the addition of mirrors or may be too distant or be too fast moving. In such instances, the environmental temperature should be used as an estimate for reflected temperature [22].

### 2.4. Other environmental parameters

Besides reflected temperature, environmental temperature, *T*_env_, and the transmissivity of the atmosphere, *τ*_atm_, are also specified in equation (2.7), and require entry into the thermographic camera. Environmental temperature allows the camera to account for the radiation emitted by the air between the camera and the target. Transmissivity of the atmosphere, *τ*_atm_, accounts for how well that radiation travels through the air between the camera and target. Transmissivity of the atmosphere is normally estimated by the camera using the distance of the target from the camera, *d*, and the percentage relative humidity of the environment, *rh*. Usually, both values are entered into the camera which then computes *τ*_atm_. Environmental temperature, environmental humidity and camera distance are easily estimated using standard measurement tools. To maximize accuracy these should also be measured simultaneously with thermography measurements, as noted in table 1. However, *τ*_atm_ is typically very close to 1 [23,24]. Consequently, the effects of changes in these parameters are normally very small. In most instances, the accuracy in these measurements has little effect on thermography data. Therefore, these parameters are often not measured alongside each thermograph, and an appropriate value is chosen for calculations [23]. Such practices have the advantage of saving time with minimal effects on accuracy. The potential exceptions to this are in extreme scenarios such as very hot or humid environments, or where measurements are being taken over a long distance. In such cases, these inputs should be measured.

## 3. Impacts of parameter omission

Above, we have discussed the thermographic parameters needed to accurately estimate temperature using thermal cameras, and the relative importance of the values chosen for these parameters. Emissivity estimated from the same kinds of tissue can vary [44,50], which means that the chosen emissivity value will have a drastic impact on the accuracy of thermographic measurements. Accuracy of measurement is also affected by the extent to which reflected temperature and other environmental parameters are accounted for [24]: whether they are measured; if so how they are measured; and, if not, what value was assumed for calculations. For this reason, when thermographic temperature measurements are made, the values used for emissivity should be included in reports as a minimum standard for accurate reporting, preferably alongside the method by which reflected temperature was accounted for. Assuming that thermography has been carried out correctly, the failure to provide this parameter information represents an incomplete methodology, and potentially misrepresents the accuracy of the thermographic measurements made. This limits the reader's ability to evaluate the choice of parameters, and compromises comparable replicate studies, as experimenters repeating a methodology will need to make an increasing number of assumptions about the methodologies of previous studies. Such assumptions may include: the value of emissivity used in estimates and if or how environmental parameters were monitored and adjusted for. If environmental parameters like *T*_ref_, *T*_env_, *rh* and *d* were not adjusted during the experiment, repeat experimenters will also have to assume the values used for calculations if they are not provided. This need to assume parameter choices will impact on the usefulness of studies where the replication of the described methods is expected. These include standardized monitoring studies such as those screening injury [14], disease [51–53] or stress [12,13,32,54–57].

We assessed the frequency in which key thermography parameters are reported in the recent primary biological literature, through a systematic literature review, aiming to evaluate how well thermography is understood and reported by biologists. A lack of inclusion of thermography parameters could be the result of two different scenarios. Firstly, the thermographic camera was used correctly, with parameters adjusted appropriately, but the detail of their adjustment was not provided in the published methodology. Alternatively, the thermographic camera could have been used incorrectly, and consequently, parameters are not adjusted or reported. Thus, a lack of information on the thermography parameters, especially emissivity, could indicate that thermography is not well understood by experimenters at some level.

## 4. Methods

### 4.1. Search criteria

Our literature search was carried out using the *Web of Science* core collection (Clarivate Analytics), limited to papers published between 2007 and 2017, with the final search taking place on 17th December 2017. This comparably recent search was chosen to allow us to focus our assessments of how biologists are using thermography currently, and to minimize the effects changes in the technology might have on the reporting of methods and applications. The following search terms were used: ‘[infrared OR infra-red OR infra red] AND [thermograph* OR thermal imag* OR camera]' (‘*' denoting derivations of the word, so ‘thermal imag*’ includes derivations such as ‘thermal image' and ‘thermal imaging’).

The search was then refined further to include only publications in at least one of the following 23 Web of Science Categories: *agriculture dairy animal science*, *agriculture multidisciplinary*, *agronomy*, *behavioural sciences*, *biology*, *biophysics*, *ecology*, *entomology*, *evolutionary biology*, *fisheries*, *forestry*, *horticulture*, *marine-freshwater biology*, *ornithology*, *physiology*, *plant sciences*, *psychology*, *psychology applied*, *psychology biological*, *psychology experimental*, *psychology multidisciplinary*, *veterinary sciences* and *zoology*. Full texts of all search results were searched for using University of Bristol library subscriptions and through *Google Scholar*. If publications could still not be found, and the paper could not be excluded based on the information in the abstract provided by *Web of Science* or linked sites alone (see exclusion criteria), the corresponding authors (where contact details provided) were contacted for copies of publications. Any publication that was not obtained through these methods was excluded. A summary of the *Web of Science* search history used in our literature search can be found in electronic supplementary material, S1.

### 4.2. Review process

Search results were examined in a chronological order by a biological scientist and qualified thermographer (M.J.M.H, Level 1 thermographer, IR training centre, awarded June 2015). Publications were checked for any criteria for exclusion (criteria detailed below), a process which left only primary biological science research papers that reported work using IR thermography in some way. These papers' methodology, how thermographic tools were employed, and the inclusion of thermographic parameters were assessed. Non-English language journals were assessed with aid of a native speaking translator if the journal could not be excluded based on the abstract alone (12 papers in total, translators are listed in acknowledgements). After completion of the full review process, all search results were worked through and assessed a second time to ensure confidence and consistency in our assessment.

### 4.3. Exclusion criteria

The search criteria used in this systematic review was deliberately broad to allow for the many ways thermal cameras might be described in publications, such as ‘thermal camera’ and ‘infrared camera'. This was done to minimize the chance of accidently excluding papers that genuinely use IR thermography. This accidental exclusion of relevant papers has been identified as a major issue in systematic reviews [58]. This has the consequence that many publications included in the *Web of Science* search results, were not primary biological science papers that used thermal imaging. The exclusion criteria applied to our search results are summarized in table 2.

**Table 2.** A summary of the exclusion criteria applied to the results of our *Web of Science* search results. Each criterion for exclusion is given in the order they are applied. For each criterion, the publications that are still included, and those that are excluded, when the criteria are applied, are summarized. Also summarized here are the papers excluded from our analysis of emissivity reporting after the thermography methods assessment.

order	exclusion criterion	included in assessment	excluded on this criterion
*excluded from thermography methods assessment*			
1	not thermography	Publications that carry out thermography or images collected by infrared thermography.	Publications that do not use thermography in any way also excluded are theoretical studies on applications of thermography if studies do not make thermal imaging measurements.
2	not biological	Thermography is applied to a biological research application.	Thermography is applied to a non-biological application.
3	isolated abstract	Publications that are not isolated abstracts from conferences. Conference reports are retained if they have a methods section.	Isolated abstracts from conferences which have no featured section for reporting methods.
4	retracted article	Publications that have not been retracted by the publishing body at time of last search.	Articles that had been retracted by the publishing body for any reason at time of last search.
5	review	Article is a primary research paper.	Publication is a secondary research paper reporting or providing commentary on the findings of previous work (these publications are filed separately in electronic supplementary material, S2 for ease of reference).
*excluded from statistical analysis after thermography assessment completed*			
6	quantitative–qualitative	Paper presents temperature measurements dependent on thermography or data that required thermographic temperature measurements for its calculation. Thus, should report thermography parameter information.	Paper uses thermal imaging in an application that does not involve measuring temperature and is dependent wholly on apparent temperature. Thus, reporting of thermography parameter information is not required to assess accuracy or repeat methods.

Only publications which carried out thermography and reported data or images collected by IR thermography were included in our review, everything else was excluded as ‘not thermography'. These excluded works included those using non-thermal IR technologies, such as triggers and sensors [59–63], IR reflectance cameras [64–66], hyperspectral cameras [67] and the use of non-thermal IR devices for night vision [68–71]. Additionally, publications using ‘infrared thermometry' [72–74] as opposed to thermography were excluded (although IR thermometry tools do use the same principles for point measurements). Theoretical studies investigating applications of IR thermography [75–78], if such studies did not report any thermal imaging measurements, were also excluded.

This review aims to assess the use of IR cameras in the life sciences area. Thus, if the application of IR thermography did not appear to be biological, publications were also excluded as ‘not biological'. Such application treated as non-biological included the industrial preparation of baked goods [79], materials science [80–82], biomechanical surgery tool maintenance [83], assessment of building materials in agricultural management [84] and canal upkeep [85]. Biomechanical studies where temperatures of artificial replacements were only monitored outside the body, for example, in mechanical stress assessment [86], and studies where biological tissue mimics were employed instead of real biological targets [87,88] were likewise excluded as ‘not biological'.

Any isolated abstracts from conferences were excluded, as such summary articles typically do not normally provide detailed information on their methodology. Published conference reports were not excluded if they featured a methods section. Any retracted articles, at time of the search, were also excluded.

Lastly, review articles that either discussed IR thermography or thermography-dependent results were excluded, albeit for reference review articles were filed separately from other exclusions (see electronic supplementary material, S2).

### 4.4. Thermography methods assessment

Included publications were assessed to obtain data on how IR thermography was used and has been reported. The information extracted from each publication can be found in table 3. It was beyond the scope of this review to evaluate in each case how appropriate the parameters used were and how this influenced the value of the thermographic measurements taken within the study. This review process consequently focused on whether primary research papers provided the information needed to make such evaluations of parameter choice or repeat the study without having to assume parameter choice. For emissivity, a specific value used in measurements was required. Simply an acknowledgement that emissivity was input was deemed as insufficient as the actual value is needed for appraisal of papers. For environmental thermography parameters (*T*_ref_, *T*_env_, *rh* and *d*), indication that these were used in calculations was required. The method of *T*_ref_ measurement was also monitored, and could either be a single quoted value used for the parameter at measurements or a continuous measurement alongside the thermography measurements, as both are acceptable [23]. The information listed in table 3 could be provided at any point in the paper main text, including within thermograph figures when information was not given in the text. The article main text was the focus of the publication search, and ‘supplementary' or ‘supplemental' text was only consulted for this information if the publication explicitly directed us to do so.

**Table 3.** The information extracted from each publication during the thermography methods assessment. Each datapoint, the format of this datapoint and a description of this datapoint are given.

datapoint	format	description
thermography target	category	The subject for the research involving thermography.
quantitative temperature values	Boolean y/n	Whether the paper used thermal imaging for a qualitative or quantitative study. ‘y' if quantitative, ‘n’ if qualitative.
emissivity, *ɛ*, value given	Boolean y/n	An indicator of whether the *ɛ* value(s) used are given in the publication.
*ɛ* value(s)	value	The *ɛ* value(s) used in the study. *n/a* if the ɛ value(s) are not given.
*ɛ* value(s) measured or referenced	category	An indicator of the source for the *ɛ* value(s) used. If emissivity was measured by the researchers this is indicated here. If emissivity was taken from an existing source that source is indicated. *n* if *ɛ* value(s) are given but no indication of measurement or source is given, *n/a* if the *ɛ* value(s) are not given.
*T*_ref_ considered	Boolean y/n	An indicator that the publication accounts for reflected temperature (*T*_ref_) in temperature measurements in any way. *n* if this information is not given or if the study merely attempted to minimize reflection.
*T*_ref_ method	category	How reflected temperature (*T*_ref_) was accounted for. If the reflected temperature value is assumed to be ambient this method is listed as ‘assumed to be ambient’. *n* if publication gives a value for reflected temperature but gives no detail. *n/a* if reflected temperature is not accounted for.
*T*_env_ considered	Boolean y/n	An indicator of whether the environmental temperature was measured or estimated alongside thermal imaging.
*rh* considered	Boolean y/n	An indicator of whether environmental relative humidity was measured or estimated alongside thermal imaging.
*d* considered	Boolean y/n	An indicator of whether camera distance was measured or estimated alongside thermal imaging
camera manufacturer model	category	The manufacturer and model of the thermal camera(s) used in the publication. *n* if this information is not given.

Throughout the review, we aimed to give authors the benefit of the doubt where possible. If a study indicated at any point in the paper that the environmental factors (*T*_env_, *rh* and *d*) in the sampling area were known, it was assumed they were input into the camera. This could be simply mentioning that these parameters were measured in the thermography sampling area. If the camera was mounted in a fixed position relative to the target it was assumed that distance had been measured and input. As several thermography parameters can be referred to by various names (listed previously), any of these were acceptable. As reflected temperature, *T*_ref_, is sometimes referred to as ‘ambient temperature' [46–49], if a study referred to environmental temperature as ‘ambient temperature' it was assumed that this value was also used for reflected temperature unless stated otherwise. However, a note was made of instances where this assumption was made (table 3). For each piece of information noted in table 3, page locations within the relevant publication were noted in each publication (using the page numbers on the version accessed).

Not all applications of thermal cameras involve measurements of temperature, for example, thermal cameras can be used to spot animals at long distances or in the dark [33,89–91]. In such non-quantitative or ‘qualitative’ applications, data are dependent only on apparent temperature [24]. Consequently, thermography parameter information is not required to assess accuracy or repeat methods of qualitative studies. It is thus important in our assessment of biological thermography publications to evaluate whether thermal imaging was used in a quantitative manner or not (table 3), as this will determine whether failing to report parameters affects study accuracy or repeatability. A publication was determined to be a quantitative study if it presented temperature data dependent on thermal imaging. This thermography-dependent temperature data could be presented graphically, or as quoted temperature values, or as a thermograph with temperature scales. If the paper presented data that required temperature measurements for its calculation, such as plant water stress index [32,54,56], such papers were viewed as quantitative. Studies deemed qualitative use IR thermal imaging but do not measure temperature values.

Each paper was assigned a biological field based on the subject of research in each study. These biological fields are listed in table 4. This also allowed assessment of whether certain biological research disciplines are more likely to fail to report IR thermography parameters when they are required (in quantitative studies). The number of quantitative studies that failed and succeeded in reporting emissivity, the minimum level of parameter reporting for thermographic temperature measurements (see above), was calculated for each biological field. The association between emissivity reporting and biological field was assessed using a χ^2^ test using R v. 3.4.1 [92]. It was deemed acceptable for wholly qualitative studies to not include parameter information [24]; thus, any qualitative publications were not included in this analysis (as described in table 2).

**Table 4.** The biological fields assigned to papers based on the subject of the thermography research. A description of the research subjects of papers in each field is also provided.

biological field	thermography research subjects
agricultural animals	animals used in agricultural practice, such as cows, goats, sheep and pigs
birds and poultry	birds and poultry, includes chickens, turkeys and their eggs
earth and soil	ground, rock or soil when measured within biological studies
humans/medical	humans, including sports science, medical and psychological studies
insects	any insects
mammals	mammals, excluding humans and agricultural animals
plants	any plants, including crop science
reptiles and amphibians	any reptiles and amphibians
other	any subject not covered in the above biological fields

## 5. Results

**Figure 1.**
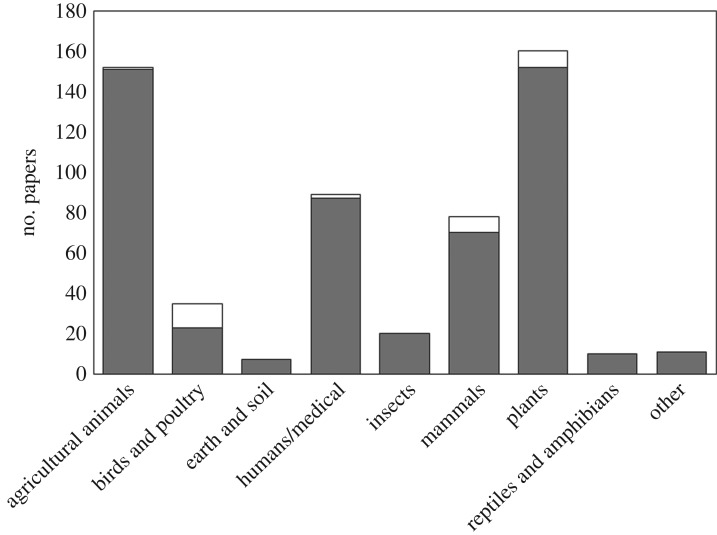
The frequency of thermography papers within each biological field, as categorized in table 4. Quantitative and qualitative papers are indicated by shading: quantitative papers, darker grey shading; qualitative papers, clear white.

The search yielded a total of 1219 search results. Exclusions accounted for most of this number. 575 publications were excluded in total: 466 ‘not thermography'; 35 ‘not biological’; 36 isolated abstracts; 1 retracted; and 37 that were not obtained by the authors and could not be otherwise excluded. This left 562 primary biological publications which employed IR thermography and a further 82 reviews featuring IR thermography. Of these 562 primary publications, 531 (94.48%) were deemed to use quantitative temperature measurements in some way, leaving 31 (5.52%) wholly qualitative studies. The frequency of quantitative and qualitative papers in each biological field is presented in figure 1.

**Figure 2.**
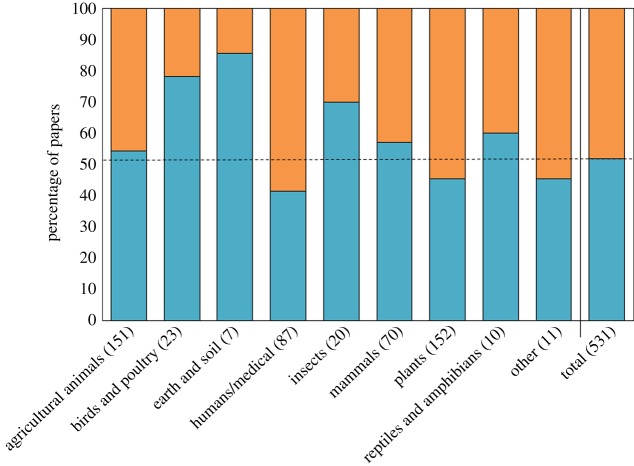
The percentage reporting of emissivity within all quantitative papers (total) and different biological fields, as categorized in table 4. Lower blue bars indicate the percentage of papers that report emissivity, higher orange bars indicate the percentage of papers that fail to report emissivity. The dotted line indicates 51.98%, the percentage of all quantitative papers that report emissivity, allowing comparison of how frequency of reporting differs compared to the overall frequency. Numbers of quantitative papers in each biological field and the total are indicated in brackets.

Of the 531 quantitative papers, where camera parameter inputs are necessary for accurate temperature measurements, 52.0% of all quantitative studies provided emissivity values (276 publications) and 48.0% of all quantitative studies failed to provide emissivity values (255 publications). Figure 2 shows the percentage of quantitative papers in each biological field that report emissivity compared to all quantitative papers. χ^2^ analysis revealed a significant association between the biological field and reporting of emissivity ($X_{8}^{2}=20.235$, *p* = 0.01). This association is largely due to papers in the ‘birds and poultry', ‘insects' and ‘earth and soil' biological fields reporting emissivity more frequently than expected and the ‘plants’ and ‘humans/medical' biological fields reporting emissivity less frequently than expected. Table 5 gives the frequencies of emissivity reporting across research fields alongside expected frequencies and Pearson residual used in our χ^2^ analysis. Of the 276 papers that provided emissivity values, 45.2% (126 publications) provided a source for that value choice and a further 5.4% (15 publications) measured the value within the study. A summary of emissivity values used in studies measuring similar targets, targets of the same research field, is given in table 6.

**Table 5.** The realized, expected and Pearson residual values of emissivity reporting used in *χ*² analysis of emissivity reporting within each biological field (biological field described in table 4). Realized frequency represents the actual observed values of emissivity reporting. Expected frequency represents the frequency of reporting expected if no effect of research field was present, given the size of the groups. Pearson residual values indicate the relative influence of the research field on a *χ*² analysis result.

	realized frequency		expected frequency		Pearson residuals	
	emissivity value not given	emissivity value given	emissivity value not given	emissivity value given	emissivity value not given	emissivity value given
agricultural animals	69	82	73	78	−0.413	0.397
birds and poultry	5	18	11	12	−1.819	1.748
earth and soil	1	6	3	4	−1.288	1.238
humans/medical	51	36	42	45	1.426	−1.371
insects	6	14	10	10	−1.163	1.118
mammals	30	40	34	36	−0.624	0.599
plants	83	69	73	79	1.171	−1.126
reptiles and amphibians	4	6	5	5	−0.366	0.352
other	6	5	5	6	0.312	−0.300

**Table 6.** A summary of emissivity values reported by publications monitoring similar biological targets, targets of the same research field and for all studies.

	agricultural animals	birds and poultry	earth and soil	humans/ medical	insects	mammals	plants	reptiles and amphibians	other	total
mean *ɛ* value	0.969	0.956	0.978	0.976	0.964	0.977	0.957	0.970	0.947	0.967
standard deviation in *ɛ*	0.018	0.033	0.020	0.007	0.009	0.015	0.038	0.020	0.059	0.026
minimum *ɛ* value	0.92	0.86	0.95	0.95	0.95	0.95	0.8	0.95	0.85	0.8
maximum *ɛ* value	1	1	1	0.98	0.97	1	1	1	1	1

Reflected temperature was only reported in 26.7% (142 publications) of all quantitative papers. Within papers that gave emissivity values, reflected temperature was reported in 41.7% (115 publications) of papers. However, in 52.2% (60 publications) of these papers reflected temperature information was not explicitly given but ‘assumed to be ambient'. In papers that failed to give emissivity, reflected temperature was reported in only 10.6% of papers (27 publications).

Environmental parameters associated less directly with IR thermography tended to be reported more frequently than emissivity and reflected temperature. With environmental temperature, environmental humidity and camera distance being reported in 81.2%, 51.6% and 66.7% of all quantitative papers respectively. Environmental temperature, environmental humidity and camera distance were reported more frequently in papers that gave emissivity values (89.9%, 60.9% and 80.1%, respectively) than those that did not (71.8%, 41.6% and 52.2%, respectively), but this difference between papers that report emissivity and those that did not was less stark than that seen in reflected temperature.

A list of the 1219 papers found in our search categorized into primary papers, reviews and exclusions as well as the data extracted from each paper can be found in electronic supplementary material, S2. A summary of the frequency of parameter reporting, broken down further by biological field, can be found in table 7.

**Table 7.** A breakdown summary of primary thermography papers in our *Web of Science* search. Given are frequencies (and percentages where indicated) of publications at each level of our thermography reporting assessment. This is given for all primary thermography publications (total) and broken down by research field (as defined in table 4).

	agricultural animals	birds and poultry	earth and soil	humans/ medical	insects	mammals	plants	reptiles and amphibians	other	total
primary thermography papers:										
qualitative studies	1	12	0	2	0	8	8	0	0	31
quantitative studies	151	23	7	87	20	70	152	10	11	531
research field total	152	35	7	89	20	78	160	10	11	562
of all quantitative studies:										
number that:										
* ɛ* value not given	69	5	1	51	6	30	83	4	6	255
* ɛ* value given	82	18	6	36	14	40	69	6	5	276
* ɛ* value referenced or measured	25	9	2	19	13	19	47	3	3	140
* T*_ref_ measured	50	13	2	19	5	24	27	2	0	142
* T*_env_ measured	119	21	5	73	18	61	119	8	7	431
* rh* measured	90	12	3	43	8	30	83	2	3	274
* d* measured	125	13	5	48	5	51	98	3	6	354
percentage that:										
* *failed to give *ɛ*	46%	22%	14%	59%	30%	43%	55%	40%	55%	48%
* *gave *ɛ*	54%	78%	86%	41%	70%	57%	45%	60%	45%	52%
of quantitative studies that gave *ɛ*:										
* *number that:										
* T*_ref_ measured	34	11	2	17	5	19	25	2	0	115
* T*_ref_ measured but ‘assumed to be ambient'	22	10	1	10	5	7	3	2	0	60
* T*_env_ measured	72	18	5	33	12	38	61	5	4	248
* rh* measured	53	10	3	21	6	22	49	2	2	168
* d* measured	72	11	5	27	3	33	63	2	5	221
of quantitative studies that failed to give *ɛ*:										
number that:										
* T*_ref_ measured	16	2	0	2	0	5	2	0	0	27
* T*_env_ measured	47	3	0	40	6	23	58	3	3	183
* rh* measured	37	2	0	22	2	8	34	0	1	106
* d* measured	53	2	0	21	2	18	35	1	1	133

## 6. Discussion

Infrared thermography parameters are an important part of making accurate thermography measurements [22–25]. Failure to include this information represents incomplete reporting on methodologies and can compromise the value and utility of studies that depend on thermography. Furthermore, it can indicate some misunderstanding of parameter importance and the thermal imaging methods used. The systematic review of biological primary research papers presented above reveals that, of those which carried out some kind of quantitative thermographic measurements, 48% failed to give the emissivity values used. Although this significantly varied between different biological research fields, we note that a large portion of all fields failed to give any indication of emissivity. Reporting emissivity represents the minimum parameter information that quantitative papers ought to include. Reflected temperature, the other large contributor to accuracy of biological thermographic measurements, was reported less frequently than emissivity, in 26% of all quantitative papers. This value includes those where reporting was unclear but the descriptions suggest that ambient temperature was entered as reflected temperature in calculations. It appears that the true frequency of reflected temperature reporting is likely to be lower. These findings reveal biological literature to be quite poor at reporting basic thermography parameter information used in studies, and suggests that greater effort is needed on the part of authors to report key thermography parameters.

Environmental temperature (*T*_env_), relative humidity (*rh*) and camera distance (*d*) have little influence on the accuracy of temperature measurements [22,23]. Nevertheless, reporting of these environmental parameters is found more frequently than explicit statements of values for emissivity and reflected temperature. This tendency for papers to report these less critical parameters seems to be the result of two factors. Firstly, we assumed in our analysis that if these parameters were known they were entered into the camera. Secondly, there is often a biological reason to include monitoring of these environmental factors independent of their influence on thermography. This is especially true of environmental temperature, a key biological variable [1–8]. This means even without any knowledge of what parameters needed to be entered into the camera, and included in the report, it is likely authors would have monitored and reported these environmental parameters. This explains why many papers that failed to give emissivity and reflected temperature still gave environmental temperature and humidity (table 7). This, unfortunately, suggests the high inclusion frequency of these parameters is not indicative of understanding of thermography.

Without parameter information it is difficult to assess the accuracy of thermographic measurements within papers, or to tell if thermography was carried out correctly or not. A number of studies (10.6%) appear to give information on reflected temperature when emissivity information is not given [93–95], or mention that emissivity was input into the camera [53,93,96,97] or even measured [98] but provide no information on the value used. These suggest an understanding of thermography and the parameters involved, most probably indicating correct operation of thermal cameras but with incomplete reporting. However, many quantitative studies make use of thermal cameras but make no mention of emissivity or reflected temperature at all [55,57,99–101]. Camera models and sensitivities and the temperature ranges displayed in images are given but not thermography parameters. Camera specifications are useful for assessment of measurement accuracy, and at least the model of camera used should be reported. However, quoted accuracies of the camera only apply when the camera inputs are correct. Likewise, the temperature range applied to the image, while influencing the image seen by operators and in reports, does not influence the temperature measurements given [24]. Taken as a whole, the frequent failure to report thermographic parameter information is likely to be the result of a combination of both scenarios. In both cases, our ability to actually appraise the accuracy and repeatability of these studies is compromised. More worryingly, if no accounting for thermography parameters has been conducted, there is a strong possibility that these papers suffer from a larger level of inaccuracy in their measurements. As these two quite different causes of parameter omission cannot be easily distinguished and have quite different effects on the paper's validity and usefulness, it is critical that researchers report parameter information. At the very least, this will then confirm that these settings were taken into account when carrying out thermographic measurements.

We found a significant association between research field and emissivity reporting, although the level of reporting was not high in most research fields (figure 2). Research fields with a very large amount of quantitative thermography publications ‘plants' and ‘humans/medical' tended to report emissivity slightly less often than other fields, while smaller groups like ‘birds and poultry', ‘earth and soil' and ‘insects' reported emissivity more often. That said, the largest research field from our review, ‘agricultural animals', reported emissivity at about the average frequency. It is likely that existing publications, especially those in the same research field, set a precedent for authors and reviewers, that thermography parameter information does not need to be included in new publications. This may explain the lower frequency of parameter reporting in certain research fields. Such an explanation could be applied more generally to explain the low frequency of parameter reporting throughout biology. It is important that journals ask for this parameter information, at least emissivity, to be included in the future to prevent such a precedent continuing. As the research fields applied to this review are deliberately quite broad, further breakdowns of the research fields would perhaps reveal specific subdivisions more prone to parameter omission than others. However, no field reported emissivity with great frequency, with failure ranging from 20% to 60% of cases across fields. So, tendency to not include parameter information is likely to continue into subdivided fields to some extent.

While our systematic review suggests that an issue exists with thermography parameter reporting in biology, it does not necessarily give a full representation of how well biologists carry out thermography. Successfully reporting parameters such as emissivity does not guarantee thermography was carried out correctly. Other operation issues can still occur when parameter settings are input correctly. Furthermore, it was beyond the scope of our review to evaluate in each instance how applicable the values used for emissivity actually were, and instead our focus was upon whether such appraisals can be done based on the information reported. Consequently, it is possible that the values chosen were still inappropriate and result in inaccurate temperature measurements. However, most often biological tissues have an emissivity of approximately 0.9 [22,25], and this is supported by the values found in the review which range from 0.8 to 1. Although, our review confirms that estimates can vary even within similar applications (table 6). In papers where emissivity values are supported by measurements or a source which measures emissivity of the tissues thermographed, we can be more confident in the emissivity values chosen. For this reason, we strongly encourage authors to provide sources for emissivity values chosen. As certain biological targets can be hard to measure emissivity from, particularly when delicate or hard to access, papers providing information on biological tissue emissivity [38,41–44,102] should be encouraged as they will help biological thermographers make more informed parameter choices and be more precise in their measurements.

Our review treats all quantitative thermography as equally important to studies; we made no evaluation of how critical the temperature measurements were to the paper's findings (outside of assessing if the paper was qualitative and quantitative). It is possible that some papers may use thermography in such a minor way that authors felt parameter detail unnecessary. However, reporting parameter information represents a small addition to the methods. Furthermore, in such instances where the accuracy of measurements is less important, papers should still give the information on parameters, but perhaps need not worry for a precise estimate of emissivity or monitor environmental parameters with every measurement. Our review process did not penalize papers for applying these less accurate approaches if they reported the necessary information, consequently a less precise approach for less critical measurements was acceptable within our review.

Frequently, emissivity and other parameter values were provided within a thermograph figure with no mention of it in the main text [103–105]. Our review process counted this as reporting, as the information was indicative that parameters were adjusted, or at least are known. However, in such instances the value could easily be overlooked if the reader were not experienced with thermography. This is particularly likely when the thermography format is unusual, perhaps due to a less common camera manufacturer. Inclusion of parameters within the article text should be encouraged over inclusion within thermographs.

## 7. Conclusion

This study has highlighted a common tendency for biologists to omit information on critical thermographic parameters such as emissivity and reflected temperature in published primary literature. This omission suggests a lack of understanding of thermographical methods. More care should be taken to include parameter information in publications. This will improve clarity and confidence in measurements but also allow the assessment of the limitations of thermography in different types of biological studies. Fortunately, the addition of parameter information represents a small effort which can significantly improve the evaluation of reported research and awareness of the correct use of thermal cameras in biological studies. It is recommended as a minimum that the emissivity values should be given, preferably with sources or measurements supporting the parameter choice. Additionally, the method of assessing reflected temperature should be included as well.

## Supplementary Material

Supplementary material 1

## Supplementary Material

Supplementary material 2

## Data accessibility

All data are available in the electronic supplementary material.
