# Supplementary material for: Reporting of thermography parameters in biology: a systematic review of thermal imaging literature
Source: R Soc Open Sci. 2018 Dec 5;5(12):181281. doi: 10.1098/rsos.181281 (PMC6304129; doi:10.1098/rsos.181281)
Supplement: Supplementary material 1 [file rsos181281supp1.pdf]

Supplementary material S1: A summary of the search used in our systematic literature review of *Web of Science*. The full literature search was made up of several searches. Each search made is identified by the order in wich the searches were made (#1, #2 etc.). Settings applied for various *Web of Science* criteria are given within each search. Throughout all searches the WOS criteria Indexes=SCI-EXPANDED, SSCI, A&HCI, CPCI-S, CPCI-SSH and ESCI Timespan=2007-2017

#1: TOPIC=(INFRARED)

#2: TOPIC=(INFRA-RED)

#3: TOPIC=("INFRA RED")

#4: TOPIC=(THERMOGRAPH*)

#5: TOPIC=("THERMAL IMAG*")

#6: TOPIC=(CAMERA*)

#7: #3 or #2 or #1

#8: #6 or #5 or #4

#9: #8 and #7

#10: #9 Refined by publication years: (2007 OR 2008 OR 2009 OR 2010 OR 2011 OR 2012 OR 2013 OR 2014 OR 2015 OR 2016 OR 2017 OR 2018)

#11: #10 Refined by: *Web of Science* categories: (AGRICULTURE DAIRY ANIMAL SCIENCE or AGRICULTURE MULTIDISCIPLINARY or AGRONOMY or BEHAVIORAL SCIENCES or BIOLOGY or BIOPHYSICS or ECOLOGY or ENTOMOLOGY or EVOLUTIONARY BIOLOGY or FISHERIES or FORESTRY or HORTICULTURE or MARINE FRESHWATER BIOLOGY or ORNITHOLOGY or PHYSIOLOGY or PLANT SCIENCES or PSYCHOLOGY or PSYCHOLOGY APPLIED or PSYCHOLOGY BIOLOGICAL or PSYCHOLOGY EXPERIMENTAL or PSYCHOLOGY MULTIDISCIPLINARY or VETERINARY SCIENCES or ZOOLOGY)
